# Osteoprotegerin is an Early Marker of the Fibrotic Process and of Antifibrotic Treatment Responses in Ex Vivo Lung Fibrosis

**DOI:** 10.1007/s00408-024-00691-5

**Published:** 2024-04-20

**Authors:** Kurnia S. S. Putri, Adhyatmika Adhyatmika, Carian E. Boorsma, Habibie Habibie, Mitchel J. R. Ruigrok, Peter Heukels, Wim Timens, Marina H. de Jager, Wouter L. J. Hinrichs, Peter Olinga, Barbro N. Melgert

**Affiliations:** 1 Department of Pharmaceutical Technology and Biopharmacy, Groningen Research Institute for Pharmacy, University of Groningen, Groningen, The Netherlands; 2 Department of Pharmacokinetics, Toxicology and Targeting, Groningen Research Institute for Pharmacy, University of Groningen, Groningen, The Netherlands; 3 Faculty of Pharmacy, Universitas Indonesia, Depok, Indonesia; 4 Drug Targeting and Personalized Medicine Research Group, Faculty of Pharmacy, Universitas Gadjah Mada, Yogyakarta, Indonesia; 5 Faculty of Pharmacy, Hasanuddin University, Makassar, Indonesia; 6 Department of Pulmonary Medicine, Erasmus MC, Rotterdam, The Netherlands; 7 Department of Pathology and Medical Biology, University Medical Center Groningen, Groningen, The Netherlands; 8 GRIAC Research Institute, University Medical Center Groningen, Groningen, The Netherlands; 9 Department of Molecular Pharmacology, Groningen Research Institute for Pharmacy, University of Groningen, Groningen, The Netherlands

**Keywords:** TNFRSF11B, RANKL, Nintedanib, Pirfenidone, Biomarker

## Abstract

**Background:**

Lung fibrosis is a chronic lung disease with a high mortality rate with only two approved drugs (pirfenidone and nintedanib) to attenuate its progression. To date, there are no reliable biomarkers to assess fibrosis development and/or treatment effects for these two drugs. Osteoprotegerin (OPG) is used as a serum marker to diagnose liver fibrosis and we have previously shown it associates with lung fibrosis as well.

**Methods:**

Here we used murine and human precision-cut lung slices to investigate the regulation of OPG in lung tissue to elucidate whether it tracks with (early) fibrosis development and responds to antifibrotic treatment to assess its potential use as a biomarker.

**Results:**

OPG mRNA expression in murine lung slices was higher after treatment with profibrotic cytokines TGFβ1 or IL13, and closely correlated with Fn and PAI1 mRNA expression. More OPG protein was released from fibrotic human lung slices than from the control human slices and from TGFβ1 and IL13-stimulated murine lung slices compared to control murine slices. This OPG release was inhibited when murine slices were treated with pirfenidone or nintedanib. OPG release from human fibrotic lung slices was inhibited by pirfenidone treatment.

**Conclusion:**

OPG can already be detected during the early stages of fibrosis development and responds, both in early- and late-stage fibrosis, to treatment with antifibrotic drugs currently on the market for lung fibrosis. Therefore, OPG should be further investigated as a potential biomarker for lung fibrosis and a potential surrogate marker for treatment effect.

**Supplementary Information:**

The online version contains supplementary material available at 10.1007/s00408-024-00691-5.

## Introduction

Lung fibrosis is a chronic lung disease that is characterized by deposition of excessive extracellular matrix (ECM) in lung parenchyma, leading to disruption of gas exchange, organ malfunction, and ultimately death from respiratory failure [1, 2]. It has a high mortality rate with an average survival after diagnosis of about 2–3 years [3, 4]. The etiology of lung fibrosis remains unclear but likely involves repeated injury to, and therefore loss of, epithelial cells, followed by a dysregulated wound repair response involving fibroblasts and macrophages [2, 3, 5]. To date, there are no effective drugs to stop or reverse fibrosis development, but two drugs have been approved by the FDA that can attenuate progression of the disease: pirfenidone and nintedanib [6–8]. Unfortunately, the development of more and better drugs is hampered by the lack of easy-to-assess early markers of fibrosis development and treatment responses.

We recently described a novel marker called osteoprotegerin (OPG) that is highly upregulated in lung tissue of patients with lung fibrosis and appears to be involved in regulating alveolar epithelial regeneration [9, 10]. A large multicenter cohort study also found OPG to be a biomarker of progressive fibrosing interstitial lung disease [11]. OPG is also known as tumor necrosis factor receptor superfamily member 11B (TNFRSF11B) and is a decoy receptor for receptor activator for nuclear factor kappa-B ligand (RANKL) and tumor necrosis factor-related apoptosis-inducing ligand (TRAIL) [12]. The canonical function of OPG is to regulate osteogenesis and is produced by osteoblasts to control osteoclast activity [13]. However, we have additionally shown that OPG is produced and secreted by lung fibroblasts under the influence of the key profibrotic mediator transforming growth factor beta 1 (TGFβ1) [14]. Interestingly, our recent studies indicate that through its capturing of RANKL it can also inhibit alveolar epithelial repair [9, 10]. However, it is unknown if other fibrosis-associated cytokines like interleukin-13 (IL13) can induce OPG expression in lung tissue and how OPG secretion is affected by treatment with the two antifibrotic drugs currently on the market. We therefore investigated the regulation of OPG early in the process of fibrosis development with either TGFβ1 or IL13 to elucidate whether it tracks with fibrosis development and responds to antifibrotic treatments to assess its potential use as a biomarker.

## Materials and Methods

### Full Experimental Details are Available in the Data Supplement

#### Mice

Eight- to twelve-weeks old C57BL/6 male mice from Harlan (Horst, The Netherlands) were kept in cages with 12 h of light/dark cycle and received food and water ad libitum. The experiments were approved by the Institutional Animal Care and Use Committee of the University of Groningen (DEC6416AA).

### Murine Precision-Cut Lung Slices

Precision-cut lung slices were prepared according to previously published methods [15, 16]. After slicing, slices were incubated in a pre-warmed 12-well plate filled with DMEM + Glutamax medium containing 4.5 g/L D-glucose and pyruvate (Gibco® by Life Technologies, Grand Island, New York, USA) supplemented with non-essential amino acid mixture (1:100), penicillin–streptomycin, 45 µg/ml gentamycin (Gibco® by Life Technologies, Grand Island, New York, USA) and 10% fetal calf serum (FCS). After 1 h of pre-incubation at 37 °C medium was refreshed. Slices were then incubated with or without 5 ng/ml TGFβ1 or 10 ng/ml IL13 and/or 1 mM pirfenidone or 0.5 µM nintedanib. After 48 h of incubation, culture medium and slices were snap frozen into liquid nitrogen and stored at −80 °C until analysis.

### Human Lung Tissue

Fibrotic lung tissue was collected with informed consent from patients undergoing transplantation for lung fibrosis at the University Medical Center Groningen and Erasmus Medical Center Rotterdam, The Netherlands. Diagnosis of the type of lung fibrosis was made in a multidisciplinary team according to current international guidelines [17]. Control tissue came from leftover material from a patient with normal lung function post-tumor resection at UMCG. Sections of lung tissue of this patient were stained with a standard haematoxylin and eosin staining and checked for abnormalities by a lung pathologist before tissue was used. The study adhered to ethical guidelines, including the UMCG Research Code and national Code of Conduct for Health Research. The use of leftover lung tissue was exempt from consent under Dutch laws, as confirmed by the Medical Ethical Committee of UMCG. All donor materials were deidentified, ensuring anonymity. The Rotterdam protocols were approved by their Medical Ethical Committee. Patient characteristics are presented in Table 1.

**Table 1:** Characteristics of the patients whose tissue were used for preparation of precision-cut lung slices

Patient	Center	Diagnosis	Age (years)	Gender	Smoking status	FEV1 (% pred)	FVC (% pred)	DLCO (% pred)
1	EMC	NSIP	54	Male	Never	31	35	23
2	EMC	IPF	65	Male	Exsmoker	41	34	NA
3	UMCG	IPF	60	Male	Exsmoker	46	38	NA
4	EMC	IPF	65	Male	Exsmoker	68	63	44
5	UMCG	IPF	61	Male	Exsmoker	59	89	38
6	UMCG	IPF	57	Male	Never	71	67	49
7	UMCG	Control	60	Male	Exsmoker	96	112	84

Abbreviations: *EMC* Erasmus Medical Center; *UMCG* University Medical Center Groningen; *IPF* idiopathic pulmonary fibrosis; *NSIP* nonspecific interstitial pneumonia; *FEV1* forced expiratory volume in the first second; *FVC* forced vital capacity; *DLCO* diffusing capacity of the lungs for carbon monoxide; % *pred* percentage of the predicted value pre-bronchodilator

### Human Precision-Cut Lung Slices

Five mm cores of lung tissue of were embedded in agarose. Slices of 200–300 μm thickness were then prepared from these cores with a Krumdieck tissue slicer and were incubated in supplemented DMEM medium, as described for murine slices with or without 2.5 mM Pirfenidone. After 48 h of incubation, culture medium and slices were snap frozen into liquid nitrogen, and stored at −80 °C until analysis.

#### ELISA

Murine and human OPG levels in slice incubation medium were measured using ELISA (R&D Systems) according to the instructions provided by the manufacturer.

### Quantitative Real-Time PCR

Total mRNA was isolated from slices using a Maxwell® LEV simply RNA Cells/Tissue kit (Promega, Madison, WI). RNA was converted into cDNA by using a reverse transcriptase kit (Promega, Leiden, The Netherlands). Transcription levels of OPG, fibrosis-associated genes (collagen 1α1 (Col1α1), fibronectin (Fn), plasminogen activator inhibitor-1 (PAI1)) were measured using a SensiMix™ SYBR kit (Bioline, Luckenwalde, Germany) for murine samples, or Taqman (Eurogentech, Maastricht, The Netherlands) for human samples. All primers, as listed in Table 2, were obtained from Sigma-Aldrich (Zwijndrecht, The Netherlands). Murine mRNA expression was normalized against 18s as housekeeping gene, while human mRNA was normalized against GAPDH.

**Table 2:** Sequences of primers

Primer	Forward sequence	Reverse sequence	Probe
*Murine*			
18s	CTTAGAGGGACAAGTGGCG	ACGCTGAGCCAGTCAGTGTA	
Col1α1	TGACTGGAAGAGCGGAGAGT	ATCCATCGGTCATGCTCTCT	
Fn	CGGAGAGAGTGCCCCTACTA	CGATATTGGTGAATCGCAGA	
PAI1	GCCAGATTTATCATCAATGACTGGG	GGAGAGGTGCACATCTTTCTCAAAG	
OPG	ACAGTTTGCCTGGGACCAAA	CTGTGGTGAGGTTCGAGTGG	
TGFβ1	AGGGCTACCATGCCAACTTC	GTTGGACAACTGCTCCACCT	
IL13Rα2	TGAAAGTGAAGACCTATGCTTT	GACAAACTGGTACTATGAAAAT	
*Human*			
GAPDH	ACCAGGGCTGCTTTTAACTCT	GGTGCCATGGAATTTGCC	TGCCATCAATGACCCCTTCA
Col1α1	CAATCACCTGCGTACAGAACGCC	CGGCAGGGCTCGGGTTTC	CAGGTACCATGACCGAGACGTG
Fn	AGGCTTGAACCAACCTACGGATGA	GCCTAAGCACTGGCACAACAGTTT	ATGCCGTTGGAGATGAGTGGGAA
PAI1	CACGAGTCTTTCAGACCAAG	AGGCAAATGTCTTCTCTTCC	
OPG	CCTGGCACCAAAGTAAACGC	TGCTCGAAGGTGAGGTTAGC	

### Statistics

Experiments with n ≥ 8 were tested with a D’Agostino & Pearson test for normality. Non-normal data were log-transformed or analyzed like smaller datasets (n ≤ 8) using Wilcoxon or Mann Whitney tests. Normal data were tested with a Student’s t-test. Multiple group comparisons were performed using a one-way ANOVA with Holm-Sidak correction or a Friedman test with Dunn’s correction, based on data normality. Pearson’s correlation coefficient was used to determine correlations. A p-value < 0.05 was considered significant.

## Results

### More Osteoprotegerin as well as other Fibrosis-Associated Markers after TGFβ1 Stimulation as Compared to Control

**Fig. 1:**
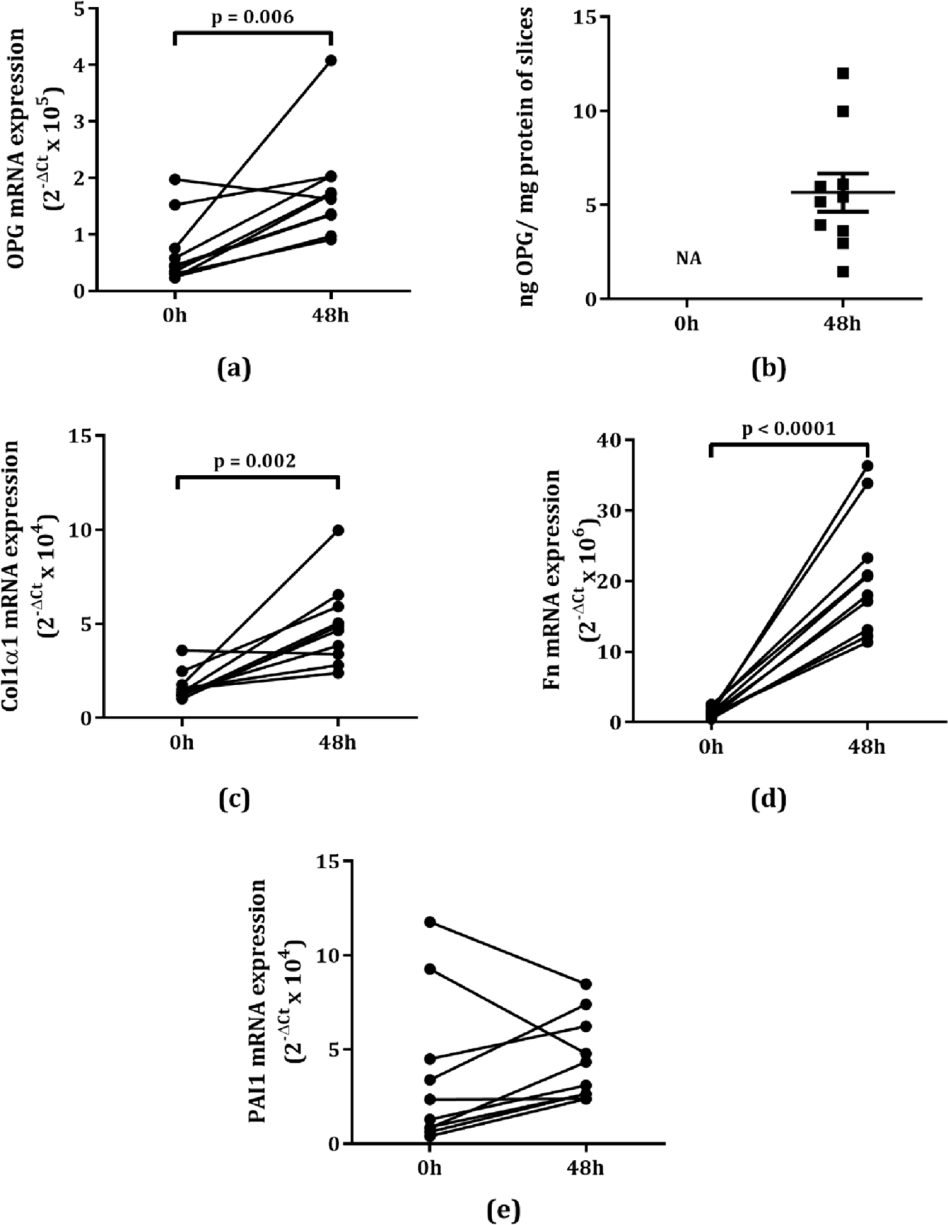
Responses of murine lung slices to incubation. Osteoprotegerin (OPG) mRNA expression was significantly higher after 48 h of incubation than freshly cut slices (**a**), and this was accompanied by higher OPG protein excretion (**b**). Collagen 1α1 (Col1α1) mRNA expression was significantly higher after 48 h of incubation as compared to freshly cut slices (**c**). The mRNA expression of fibronectin (Fn) was significantly higher after 48 h of incubation than freshly cut slices (**d**). Plasminogen activator inhibitor-1 (PAI1) expression was not higher after 48 h of incubation (**e**). Groups were compared using a Student’s t test, p < 0.05 was considered significant. NA: not applicable

To study OPG production in the early onset of fibrosis development, we incubated murine lung slices for 48 h with and without TGFβ1 and quantified OPG mRNA and protein expression levels in addition to Col1α1, Fn, and PAI1 to determine the effects of 48-h incubation and TGFβ1. We found that OPG mRNA levels in murine lung slices were significantly higher after 48 h of incubation (Fig. 1a) compared to freshly cut slices and this was accompanied by measurable levels of OPG protein excretion (Fig. 1b). Furthermore, Col1α1 and Fn mRNA levels were also higher compared to fresh lung slices, while PAI1 expression was not (Fig. 1c–e). These observations suggests that the slice model intrinsically exhibits activated wound healing processes.

**Fig. 2:**
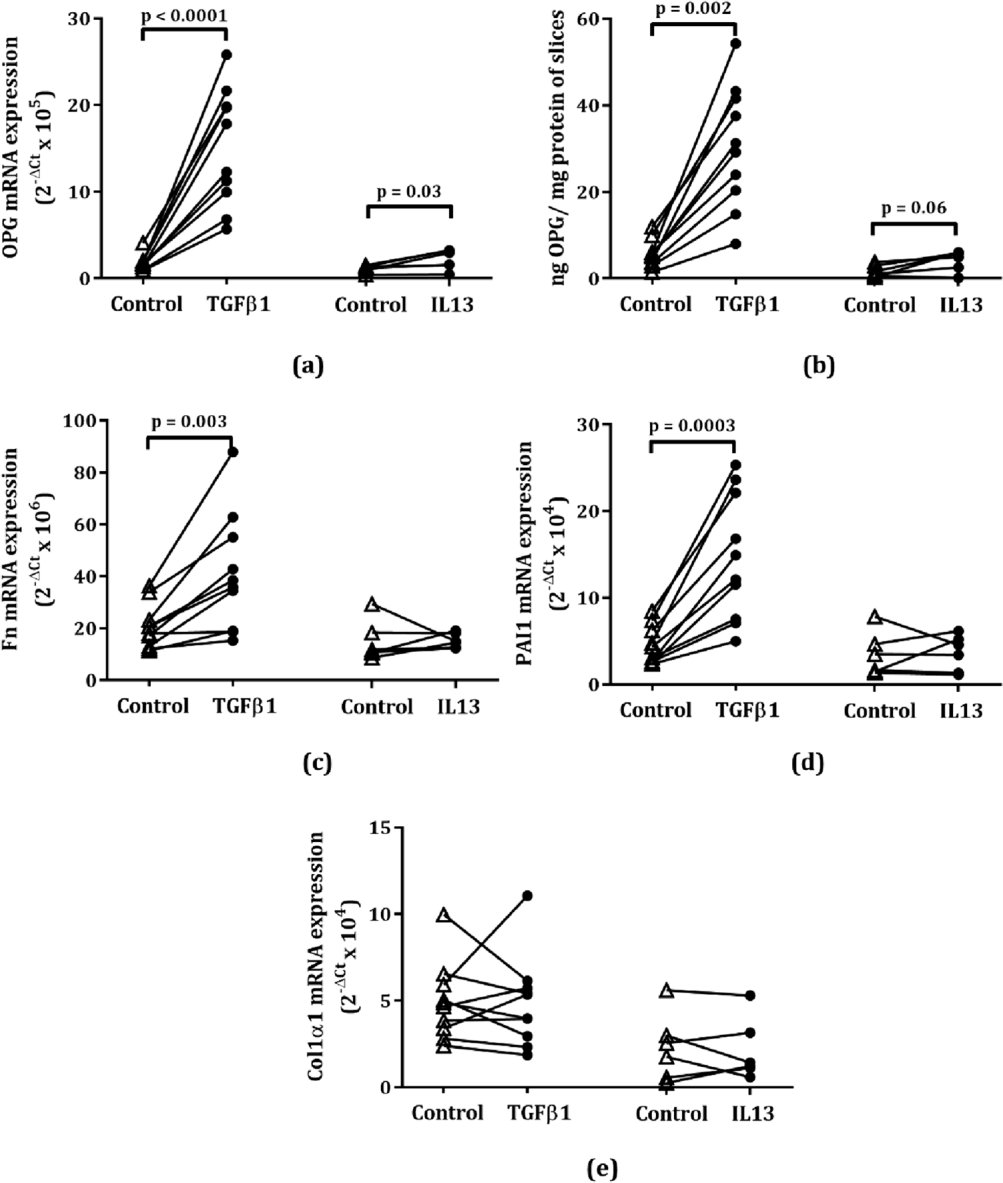
Responses of murine lung slices to treatment with TGFβ1 or IL13 for 48 h. Osteoprotegerin (*OPG*) mRNA (**a**) and protein expression (**b**) were significantly (or near-significantly) higher after TGFβ1 or IL13 stimulation. IL13 did not induce expression of fibronectin (Fn, **c**), plasminogen activator inhibitor-1 (PAI1, **d**), collagen 1a1 (Col1α1, **e**), or mRNA, while TGFβ1 induced Fn and PAI1 but not Col1α1. Groups were compared using a paired Wilcoxon test, p < 0.05 was considered significant

When giving a profibrotic stimulus by incubating slices with TGFβ1 for 48 h, we found even higher OPG mRNA levels (Fig. 2a) and protein excretion (Fig. 2b) and this corresponded with significantly higher expression of Fn (Fig. 2c) and PAI1 (Fig. 2d) compared to vehicle-treated control slices, but not of Col1α1 (Fig. 2e).

### More Osteoprotegerin after IL13 Stimulation as Compared to Control

In addition to TGFβ1, we also investigated the effect of IL13, another profibrotic cytokine, on murine lung slices. OPG mRNA and protein levels were higher after 48 h of IL13 stimulation as compared to vehicle-treated lung slices, similar to the effect of TGFβ1 on murine lung slices (Fig. 2a, b). IL13 did not induce expression of the three other fibrosis markers tested in this study, i.e. Fn, PAI1, and Col1α1 (Fig. 2c–e).

**Fig. 3:**
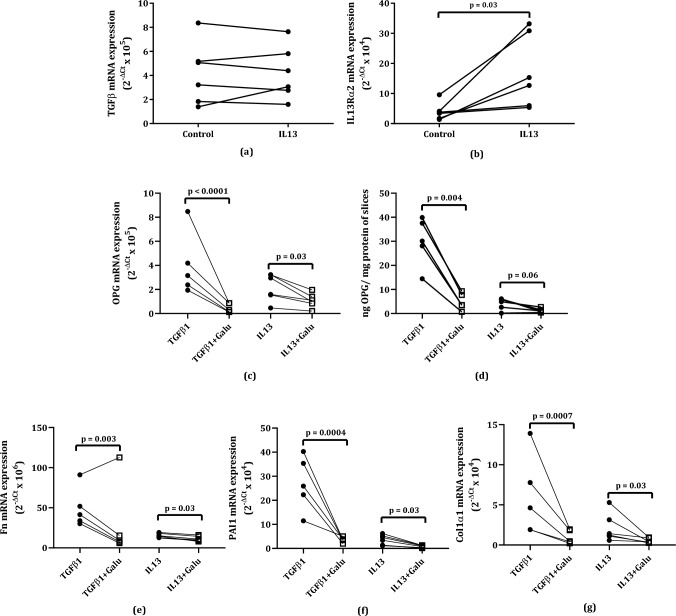
Responses of murine lung slices to IL13 stimulation and galunisertib treatment. IL13 did not lead to higher TGFβ1 mRNA expression (**a**) but did result in significantly higher interleukin 13 receptor alpha 2 mRNA expression (IL13Rα2) (**b**). Galunisertib treatment of IL13-stimulated murine lung slices resulted in significantly lower osteoprotegerin (OPG) mRNA expression (**c**) and a trend towards lower OPG protein excretion (**d**) compared to IL13 stimulation alone. Galunisertib treatment of IL13-stimulated murine lung slices also significantly lower fibronectin (Fn, **e**), plasminogen activator inhibitor-1 (PAI1, **f**), and collagen 1a1 (Col1α1, **g**) mRNA. Groups were compared using a Wilcoxon test, p < 0.05 was considered significant

To investigate the mechanism behind IL13-stimulated OPG production, we also measured mRNA expression of TGFβ1 and interleukin 13 receptor alpha 2 (IL13Rα2) in IL13-stimulated murine lung slices. We found that IL13 treatment did not lead to higher TGFβ1 mRNA expression (Fig. 3a) but did significantly induce IL13Rα2 mRNA expression (Fig. 3b).

To investigate if TGFβ1 could potentially play a role, we further treated IL13-stimulated murine lung slices with galunisertib, an inhibitor of TGFβ receptor 1 kinase. TGFβ1-stimulated slices were added as a positive control for the effect of galunisertib. We found significantly lower OPG mRNA expression and a trend towards lower OPG protein excretion compared to nontreated IL13-stimulated murine lung slices (Fig. 3c, d), in addition to lower expression of Fn, PAI1, and Col1α1 (Fig. 3e–g**)**. Incidentally, when treating unstimulated slices with galunisertib for 48 h, we also found significant lower expression of most fibrotic markers, again indicating the intrinsically activated wound healing in slices (**supplemental **Figure S1**a–e**).

### Osteoprotegerin Expression Strongly Correlates with Fn and PAI1 Expression in TGFβ1-Stimulated Murine Lung Slices and Less so in IL13-Stimulated Slices

To investigate whether expression of OPG correlates with other fibrosis markers, we compared OPG mRNA expression with its protein secretion and expression of each of the other fibrosis marker from the same experiments.

For both TGFβ1 and IL13, we found strong positive correlations between OPG mRNA expression and secreted OPG protein in culture medium (Table 3, **Supplemental **Figure S2**a, e**), while for Fn (**S2b, f)** and PAI (**S2c, g**) only TGFβ1-induced OPG mRNA correlated significantly. Col1α1 mRNA expression did not correlate with OPG mRNA after either treatment (**S2d, h**).

**Table 3:** Correlation between OPG mRNA expression with fibrosis-associated markers in in TGFβ1- or IL13-stimulated mouse lung slices

**Fibrosis-stimulating cytokine**	**Fibrosis-associated markers**	**OPG mRNA expression**	
		**Pearson correlation coefficient (r)**	**Correlation p-value**
TGFβ1	OPG protein excretion	0.79	< 0.0001
	PAI1 mRNA	0.87	< 0.0001
	Fn mRNA	0.76	0.0001
	Col1α1 mRNA	0.1	0.68
IL13	OPG protein excretion	0.79	0.002
	PAI1 mRNA	0.54	0.07
	Fn mRNA	0.44	0.16
	Col1α1 mRNA	−0.37	0.24

### More Osteoprotegerin was Released from Slices of Human Fibrotic Lung than from Control Lung Tissue, and its Release Responds to Antifibrotic Treatment

**Fig. 4:**
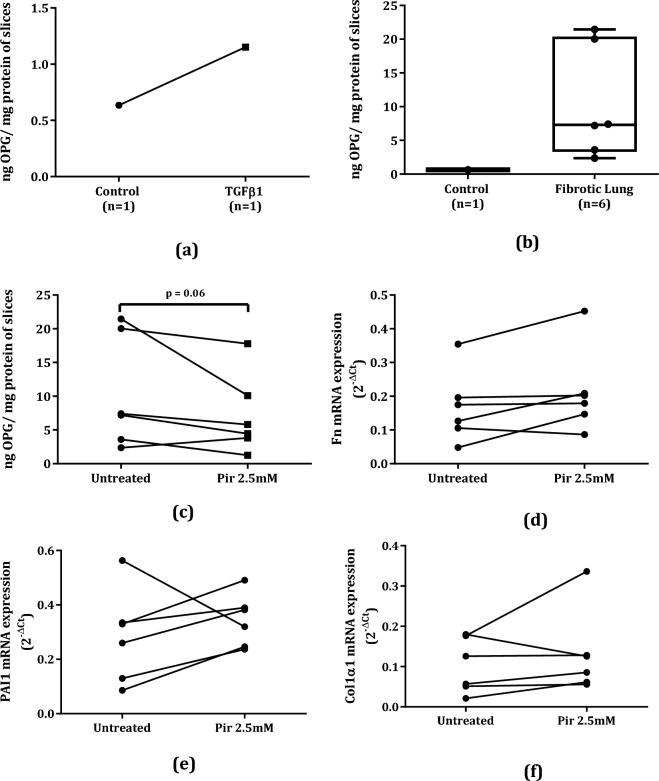
More osteoprotegerin was released from TGFβ1-stimulated human control lung slices than from the unstimulated slices, n = 1 (**a**). After 48 h of incubation, human fibrotic lung slices released more OPG than the human control lung slices (**b**). Pirfenidone treatment resulted in (near)significant lower OPG secretion from human fibrotic lung slices (**c**), but did not affect Fn (**d**), PAI (**e**) or Col1α1 (**f**) mRNA expression significantly. Groups were compared using a paired Wilcoxon test, p < 0.05 was considered significant

Similar to our findings in murine lung slices, more OPG was released from control human lung slices that were incubated with TGFβ1 for 48 h than from untreated slices (Fig. 4a). In addition, slices made from fibrotic human lung tissue released more OPG during 48 h of incubation than slices made from control lung tissue (Fig. 4b). We also found that incubating human fibrotic lung slices with 2.5 mM pirfenidone resulted in a trend towards lower OPG protein excretion into culture medium (Fig. 4c). Interestingly, lower OPG protein excretion from pirfenidone-treated human lung slices was not accompanied by lower mRNA expression of Fn, PAI1, or Col1α1 as compared to untreated slices (Fig. 4d–f**)**. We also investigated whether expression of OPG mRNA correlated with expression of fibrosis markers Fn, PAI1, or Col1α1 and found only a trend towards a positive correlation with Fn (r = 0.53, p = 0.07) and Col1α1 (r = 0.51, p = 0.09, **Supplemental **Figure S3**a–c**).

### Osteoprotegerin Release Responds to Pirfenidone or Nintedanib Treatment in Murine Lung Slices

**Fig. 5:**
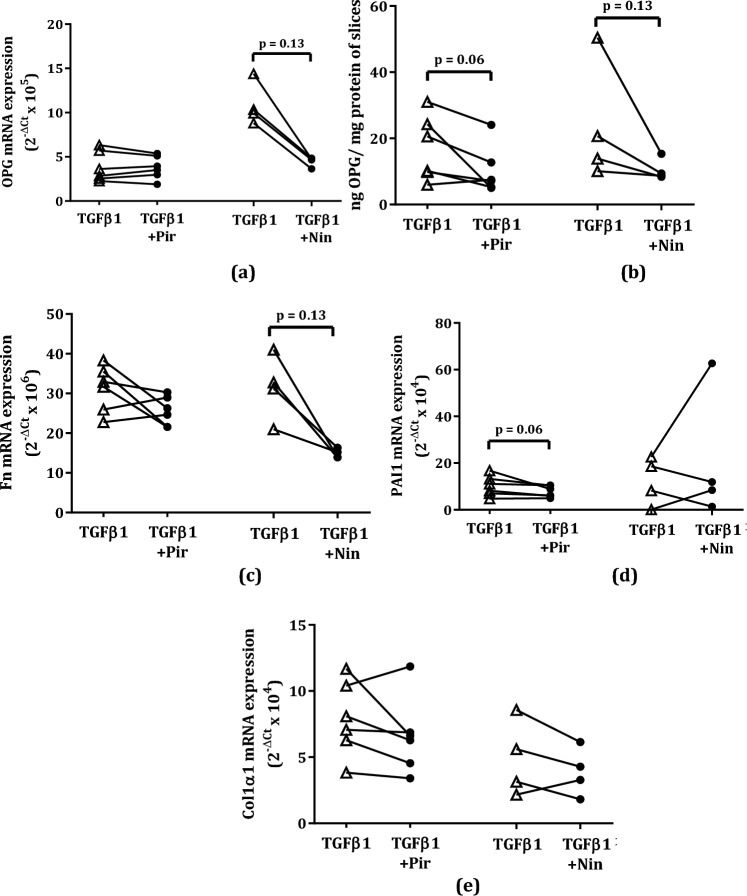
Effect of pirfenidone 1 mM or nintedanib 0.5 µM on OPG protein excretion and several fibrosis-associated markers in TGFβ1-stimulated murine lung slices. OPG mRNA was inhibited by nintedanib (Nin) but not pirfenidone (Pir) (**a**), while OPG protein release was was lower in either pirfenidone or nintedanib -treated mouse lung slices (**b**). This was accompanied by lower Fn (**c**) mRNA expression in nintedanib-treated mouse lung slices, while pirfenidone-treated mouse lung slices were only had lower PAI1 mRNA expression (**d**). Pirfenidone or nintedanib treatment exhibited no effects on Col1α1 mRNA expression (**e**) on TGFβ1-stimulated murine lung slices. Groups were compared using a Wilcoxon test, p < 0.05 was considered significant

Next, we investigated whether OPG release from TGFβ1-stimulated murine lung slices would also respond to antifibrotic treatment and found that treatment with either pirfenidone or nintedanib resulted in a trend towards lower OPG expression (Fig. 5a, b). Lower mRNA expression of OPG (Fig. 5a**)** was accompanied by lower levels of OPG protein released from pirfenidone/nintedanib-treated murine lung slices. Fn mRNA expression also showed a trend towards lower levels as compared to lung slices only stimulated with TGFβ1, but only after nintedanib treatment **(**Fig. 5c**)**. Levels of PAI1 or Col1α1 mRNA expression were not affected by the antifibrotics (Fig. 5d, e).

## Discussion

Our study investigated if OPG expression would associate with fibrotic responses induced by profibrotic cytokines TGFβ1 or IL13 in lung tissue and whether it would respond to antifibrotic treatment. OPG was initially recognized for its role in bone turnover in which it prevents bone resorption and stimulates production of extracellular matrix in cartilage [18]. However, in recent years, an increasing number of studies have shown correlations between OPG and several fibrotic conditions including liver, vascular, cardiac, kidney and intestinal fibrosis [19–29]. This study, and our own previous studies [9, 10] have now shown that this also appears to be the case for fibrosis in lung tissue and even wound healing. Our data shows that after the slicing procedure a repair or regenerative response was induced, with higher Col1α1 and Fn mRNA expression, which was accompanied by higher production of OPG. Inducing a more fibrotic response by treating with TGFβ1 magnified this inherent repair response induced by slicing.

How OPG actually influences wound repair and fibrosis is still an open question, but a study by Hao et al. suggests an interaction between TRAIL, OPG, and collagen-producing cells [30]. Using OPG-deficient mice, they showed lower collagen deposition in heart tissue, accompanied by increased expression of TRAIL and a higher level of apoptosis in heart tissue, which suggesting an association between TRAIL, OPG, and collagen production. We have previously shown fibroblasts and myofibroblasts to be important producers of OPG [9, 14] and these cells are also key cells in collagen production [31, 32]. These combined findings indicate that OPG, as a decoy receptor for TRAIL, may protect (myo)fibroblasts from TRAIL-induced apoptosis and can thereby contribute to both physiological wound healing and fibrosis. Alternatively, OPG, serving as a decoy receptor for RANKL, might also play a role in hindering RANKL-induced epithelial repair, as previously demonstrated by us [10]. Coupled with the suppression of TRAIL-induced apoptosis of myofibroblasts, this mechanism could potentially contribute to the development of fibrosis. Future studies should be designed to investigate these interactions in more detail in the context of lung fibrosis.

Interestingly, there appears to be no close connection between OPG and collagen production as OPG mRNA expression did not correlate with Col1α1 mRNA expression, while OPG did correlate with Fn and PAI1 in our model of early-stage fibrosis. Little is known about interactions between Fn, PAI1 and OPG, however, a study by Vial et al. showed that PAI1 stimulates Fn matrix assembly by disrupting the interaction between αvβ5 and vitronectin that then stimulates activation of α5β1 integrin, which increases the rate of Fn polymerization [33]. OPG has been shown to bind to αv integrins as well and may therefore enhance production of other fibrotic markers [34].

As we have shown before, OPG expression in lung tissue can clearly be promoted by TGFβ1, [24, 29, 35, 36]. To get more insight into the regulation of OPG expression, we also studied the effect of IL13, a fibrosis-associated cytokine known to be able to increase TGFβ1 expression [14, 37]. Indeed, previous studies have shown that IL13 plays an important role in the development of lung fibrosis [38, 39]. However, unlike TGFβ1, IL13 stimulation of murine lung slices did not induce mRNA expression of Col1α1, Fn and PAI1, while it did induce production of OPG mRNA and protein. This induction appeared to be directly dependent on TGFβ1 as co-treatment with galunisertib, a TGFβ receptor-I kinase inhibitor, could completely block the OPG-inducing effect of IL13. We previously showed a similar phenomenon in liver slices, in which IL13 induced OPG expression through IL13 receptor α2 (IL13Rα2)-induced TGFβ production [14]. In the present study we did find higher expression of IL13Rα2 after treatment with IL13, but not a concomitant increase in TGFβ1 expression. This may have been caused by a difference in the kinetics of different mRNA transcripts studied. However, these results do suggest that OPG is responsive towards small changes in fibrogenesis and may thus serve as sensitive marker to observe fibrosis initiation and/or progression. Indeed, a recent multicenter cohort study by Bowman et al*.* showed that OPG particularly associated with progressive fibrosing interstitial lung disease, reinforcing this view [11].

Importantly, we found that OPG was also released by both slices from lung tissue of a patient with normal lung function as well as slices from lung tissue of patients with lung fibrosis, with the latter releasing far more than slices of control lung tissue. The OPG production by control lung tissue could also be increased by stimulating with TGFβ1, suggesting similar pathways in mice and men. Due to limited availability of lung tissue from patients with normal lung function, we only obtained one sample for this study and these studies should therefore be extended for definite conclusions. Our results, however, do confirm the possibility of studying OPG as a marker of lung fibrosis in a clinical setting. The advantage of using OPG as marker of remodeling and fibrosis over other tissue proteins is that OPG is a soluble protein that can easily be measured in blood or culture media. Serum OPG should therefore be further investigated as a marker for progression of fibrotic disease in clinical practice.

In the interest of clinical applicability of OPG as a marker for treatment effects, we further investigated whether OPG production is affected by treatment with antifibrotic drugs. We found that OPG production was indeed inhibited by both drugs, even though expression of fibrosis-associated markers were not (yet) affected. The reason for this discrepancy is unclear but as many in vitro, in vivo, and clinical studies have shown pirfenidone and nintendanib can inhibit extracellular matrix production [40–48], our results reinforce the notion that OPG may be an early marker of treatment effect before any other markers are affected.

Concluding, our study has shown that OPG is upregulated in early stages of lung fibrosis and wound repair and responds to antifibrotic treatment. As OPG can easily be measured in serum, it is an interesting candidate to further investigate as a potential biomarker for fibrotic disorders of the lung and a potential marker for treatment effect of novel antifibrotics.

### Supplementary Information

Below is the link to the electronic supplementary material.

Supplementary file1 (DOCX 270 KB)

Supplementary file2 (TIF 471 KB)

Supplementary file3 (TIF 932 KB)

Supplementary file4 (TIF 482 KB)
