# Supplementary material for: Osteoprotegerin is an Early Marker of the Fibrotic Process and of Antifibrotic Treatment Responses in Ex Vivo Lung Fibrosis
Source: Lung. 2024 Apr 20;202(3):331–42. doi: 10.1007/s00408-024-00691-5 (PMC11143060; doi:10.1007/s00408-024-00691-5)
Supplement: Supplementary file 1 — Supplementary file1 (DOCX 270 KB) [file 408_2024_691_MOESM1_ESM.docx]

**Osteoprotegerin is an early marker of the fibrotic process and of antifibrotic treatment responses in *ex vivo* lung fibrosis**

**Supplemental Data**

**Materials and Methods**

*Murine Precision-cut Lung Slices*

Eight- to twelve-week old C57BL/6 male mice from Harlan (Horst, The Netherlands) were kept in cages with a 12 hours of light/dark cycle and received food and water *ad libitum*. The experiments were approved by the Institutional Animal Care and Use Committee of the University of Groningen (DEC6416AA).

Precision-cut lung slices were prepared according to the method of Oenema and co-workers[22], with several modifications. Shortly, mice were anaesthetized with isoflurane/O_2_ (Nicholas Piramal, London, UK) and then sacrificed by exsanguination via the aorta abdominalis. The lungs were filled with 1 mL low-melting temperature agarose (1.5%) (Sigma-Aldrich, Steinheim, Germany) in 0.9% NaCl through the cannulated trachea, and the lungs were directly transferred into ice-cold University of Wisconsin organ preservation solution (UW-solution). Cores of lung tissue were made by using a biopsy-puncher with a 5-mm diameter. Slices, with a weight of about 5 mg (or thickness of 250-300 µm), were prepared from these cores with a Krumdieck tissue slicer (Alabama Research and Development, USA), which was filled with ice-cold Krebs-Henseleit Buffer supplemented with 25 mM D-glucose (Merck, Darmstadt, Germany), 25 mM NaHCO_3_ (Merck), 10 mM HEPES (MP Biomedicals, Aurora, OH), saturated with carbogen (95% O_2_/5% CO_2_) and adjusted to pH 7.4.

After slicing, slices were incubated in a pre-warmed 12-well plate which was filled with 1.3 mL DMEM + Glutamax medium containing 4.5g/L D-glucose and pyruvate (Gibco® by Life Technologies, Grand Island, New York, USA) supplemented with non-essential amino acid mixture (1:100), penicillin-streptomycin, 45 µg/ml gentamycin (Gibco® by Life Technologies, Grand Island, New York, USA) and 10% fetal calf serum (FCS). After 1 hour of pre-incubation at 37 °C in an O_2_/CO_2_-incubator (MCO-18M, Sanyo, USA) which was continuously shaken at a speed of 90 rpm and saturated with 80% O_2_ and 5% CO_2_, medium was refreshed. Slices were then incubated for 48 hours in medium with or without several cytokines to mimic the fibrotic process, and with or without antifibrotic compounds. Transforming growth factor-1 (TGFβ1, 5 ng/ml) and interleukin-13 (IL-13, 10 ng/ml) were added to investigate their influence on development of fibrosis and production of OPG, while 1 mM pirfenidone and 0.5 µM nintedanib were added as antifibrotic compounds. DMSO was used as control for antifibrotic study. Medium and treatments were refreshed after 24 hours. After 48 hours of incubation, culture medium and slices were snap frozen into liquid nitrogen, and stored at -80 °C until analysis.

*Human Precision-cut Lung Slices*

Human fibrotic lung tissue was collected with informed consent from patients with end-stage lung fibrosis undergoing lung transplantation at either the University Medical Center Groningen (UMCG, Groningen, The Netherlands) or at the Erasmus Medical Center Rotterdam (Rotterdam, The Netherlands). Control human lung tissue was obtained from material leftover following tumor resection surgery at the UMCG. In Groningen, the study was conducted in accordance to the Research Code of the UMCG, as stated on <https://umcgresearch.org/w/research-code-umcg> as well as national ethical and professional guidelines Code of Conduct for Health Research (<https://www.coreon.org/wp-content/uploads/2023/06/Code-of-Conduct-for-Health-Research-2022.pdf>). The use of left-over lung tissue in this study was not subject to Medical Research Human Subjects Act in the Netherlands, as confirmed by a statement of the Medical Ethical Committee of the University Medical Center Groningen and therefore exempt from consent (Dutch laws: Medical Treatment Agreement Act (WGBO) art 458 / GDPR art 9/ UAVG art 24). All donor material and clinical information were deidentified prior to experimental procedures, blinding any identifiable information to the investigators. In Rotterdam, the Medical Ethical Committee approved all protocols followed in that center. During preparation, lung tissue was preserved in ice-cold University of Wisconsin organ preservation solution (UW-solution). Cores of lung tissue of 5 mm were made and embedded in agarose. Slices were then prepared from these cores with a Krumdieck tissue slicer and were incubated in supplemented DMEM medium, as described for murine slices. Pirfenidone (2.5 mM) was added as antifibrotic drug. Medium and treatments were refreshed after 24 hours. After 48 hours of incubation, culture medium and slices were snap frozen into liquid nitrogen, and stored at -80 °C until analysis.

*ELISA*

Murine and human OPG levels in slice incubation medium were measured using ELISA (cat #DY459 (murine), cat #DY805 (human), R&D Systems) according to the instructions provided by the manufacturer. Total OPG in the medium was corrected for the protein content of the slices, which was measured by Lowry (BIO-rad RC DC Protein Assay, Bio Rad, Veenendaal, The Netherlands).

*Quantitative Real-time PCR*

Total mRNA was isolated from slices using a Maxwell^®^ LEV simply RNA Cells/Tissue kit (Promega, Madison, WI). Final RNA concentrations were determined using Biotek Synergy HT (Biotek^®^, Winoosku, Vermont, USA). The conversion of RNA into cDNA was performed by using a reverse transcriptase kit (Promega, Leiden, The Netherlands) in a master-cycler gradient (25°C for 10 min, 45°C for 60 min, and 95°C for 5 min). Transcription levels of OPG, fibrosis-associated genes (collagen 1α1 (Col1α1), fibronectin (Fn1), plasminogen activator inhibitor-1 (PAI-1)) were measured using a SensiMix™ SYBR kit (Bioline, Luckenwalde, Germany) for murine samples, or Taqman (Eurogentech, Maastricht, The Netherlands) for human samples, and a 7900HT Real-Time RT-PCR sequence detection system (Applied Biosystems, Bleiswijk, The Netherlands) with 45 cycles of 10 min 95°C, 15 sec at 95°C, and 25 sec at 60°C following with a dissociation stage (SYBR kit) or or with 40 cycles of 10 min at 95 °C, 15 s at 95 °C and 1 min at 60 °C (TaqMan).

Gene expression was quantified using Ct values of the genes in the SDS 2.3 software program (Applied Biosystems). mRNA expression in murine lung slices was normalized against 18s as housekeeping gene, while human lung slices were normalized against GAPDH as housekeeping gene. Gene expression is shown as normalized gene expression (2^-ΔCt^) in graphs. All primers, as listed in **Table 1**, were obtained from Sigma-Aldrich (Zwijndrecht, The Netherlands).

*Statistics*

Results are presented as box-and-whisker plots using the median and min/max whiskers including individual data points or as aligned before-after plots. All results obtained from more than 8 individual experiments were analyzed for its normality by using D’Agostino & Pearson omnibus normality test. Datasets that did not have a normal distribution were log-transformed to obtain normality and if data were still not normally distributed then nonparametric tests were used. All normally distributed results were analyzed by using a paired or unpaired Student’s t-test. All results that were not normally distributed or obtained from less than 8 individual experiments were analyzed by Wilcoxon or Mann Whitney test for paired or unpaired data, respectively. When comparing multiple groups, a parametric one-way ANOVA with Holm-Sidak correction or non-parametric paired Friedman with Dunn’s correction was performed depending on normality of the data. Correlations were assessed by calculating the Pearson correlation coefficient. A p-value lower than 0.05 (p<0.05) was considered as significant.

**Supplemental Figures**

**Supplemental figure S1.** Responses of control murine lung slices to galunisertib treatment. Galunisertib treatment of control murine lung slices for 48 hours resulted in a trend towards lower osteoprotegerin (OPG) mRNA expression (a) and significantly lower collagen 1a1 (Col1α1, b), fibronectin (Fn, c), plasminogen activator inhibitor-1 (PAI1, d), and transforming growth factor beta (TGFβ, e) mRNA expression. Groups were compared using a Wilcoxon test, p<0.05 was considered significant.

**Supplemental Figure S2**. Correlations of OPG mRNA expression with OPG protein excretion and with the expression of other fibrosis markers (Fn, PAI1, and Col1α1) in murine lung slices stimulated with TGFβ1 (a, b, c, d) and IL13 (e, f, g, h). Correlations were tested using a Pearson test and presented as log data. p<0.05 was considered significant.

**Supplemental Figure S3**. Correlations of OPG mRNA expression with the expression of other fibrosis markers: Fn (a), PAI1 (b) and Col1α1 (c) in fibrotic human lung slices. Correlations were tested using a Pearson test and presented as log data. p<0.05 was considered significant.
