# Supplementary figures and images for: Osteoprotegerin is an Early Marker of the Fibrotic Process and of Antifibrotic Treatment Responses in Ex Vivo Lung Fibrosis
Source: Lung. 2024 Apr 20;202(3):331–42. doi: 10.1007/s00408-024-00691-5 (PMC11143060; doi:10.1007/s00408-024-00691-5)

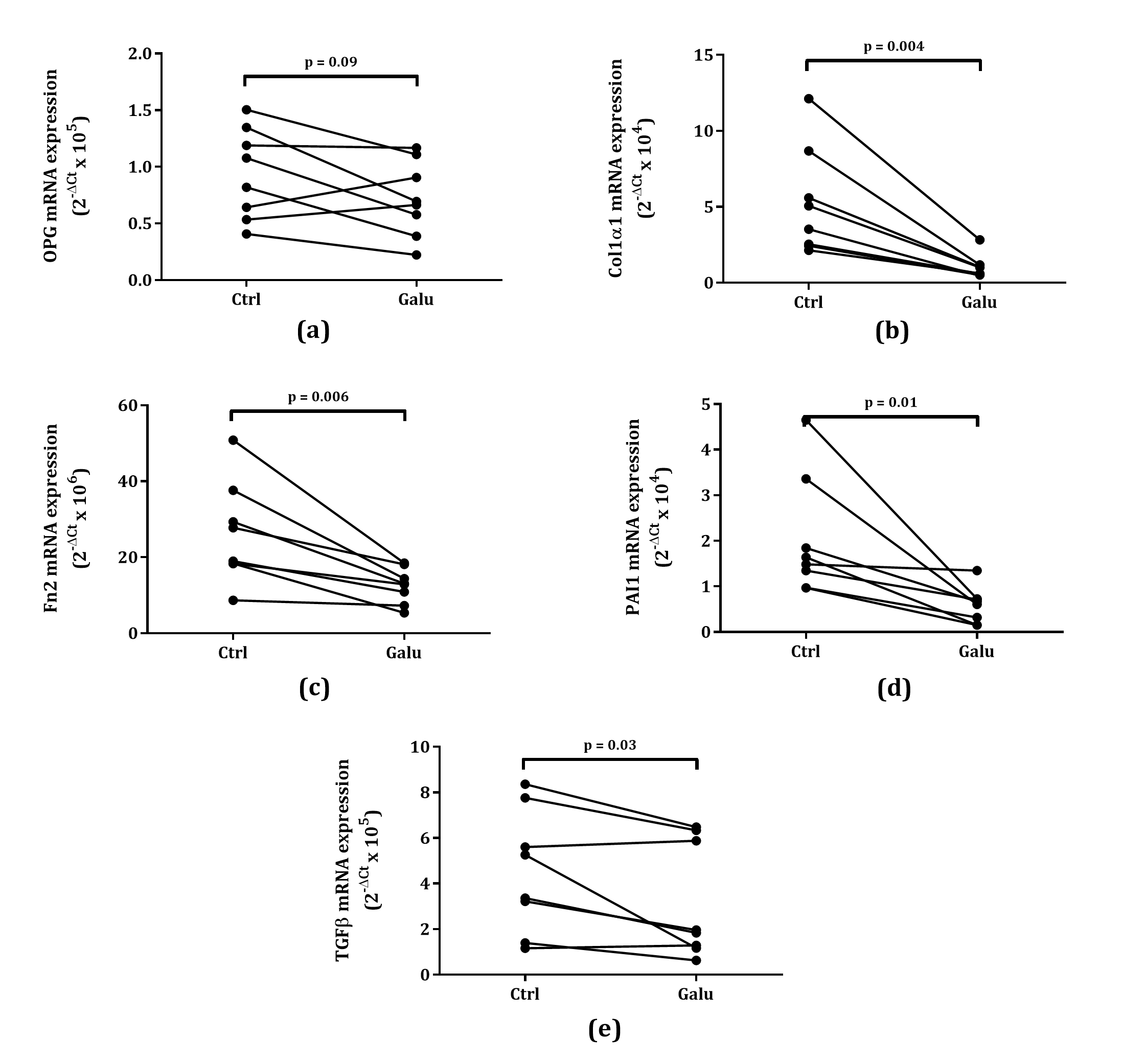

Supplement: Supplementary file 2 — Supplementary file2 (TIF 471 KB) [file 408_2024_691_MOESM2_ESM.tif]

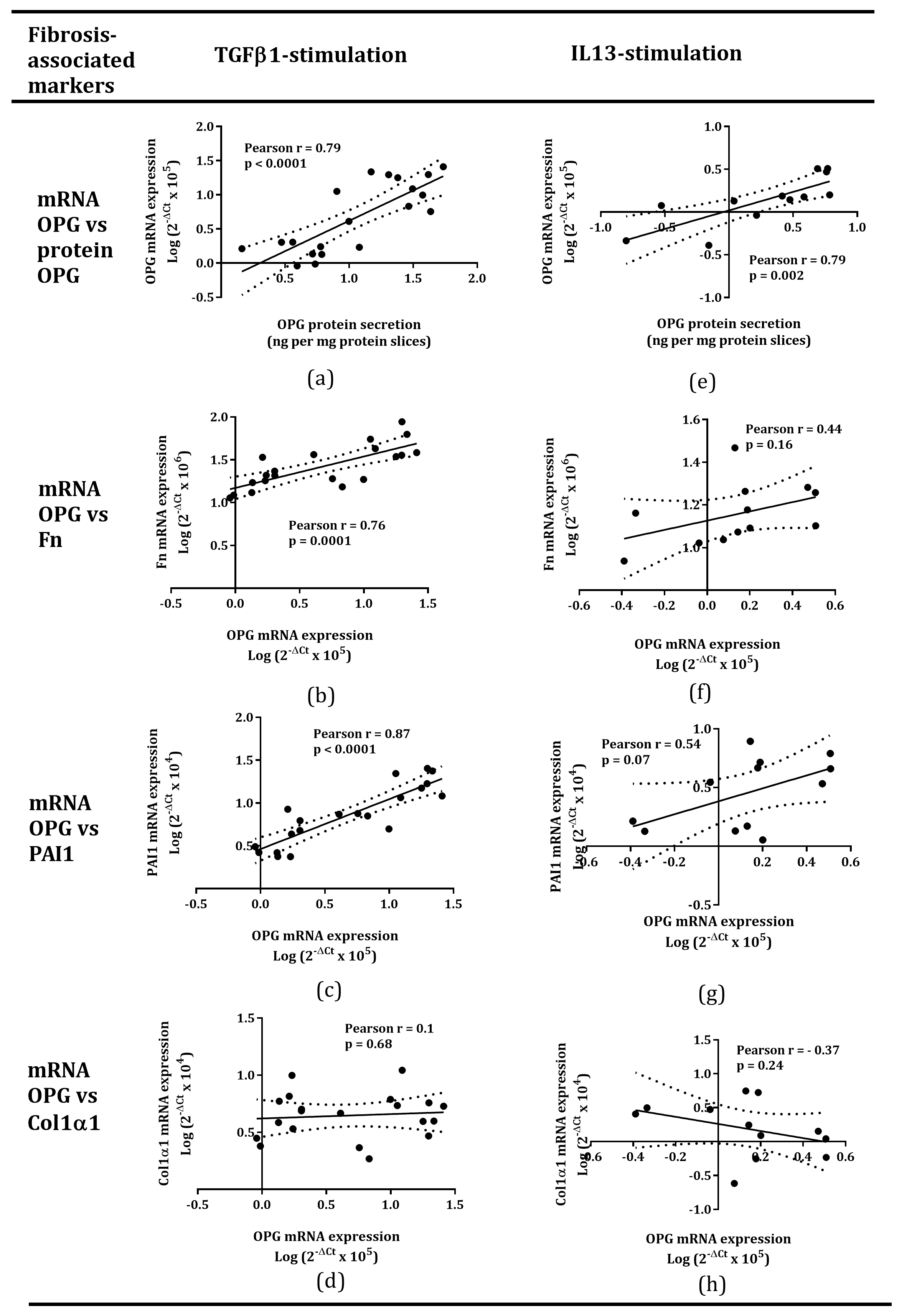

Supplement: Supplementary file 3 — Supplementary file3 (TIF 932 KB) [file 408_2024_691_MOESM3_ESM.tif]

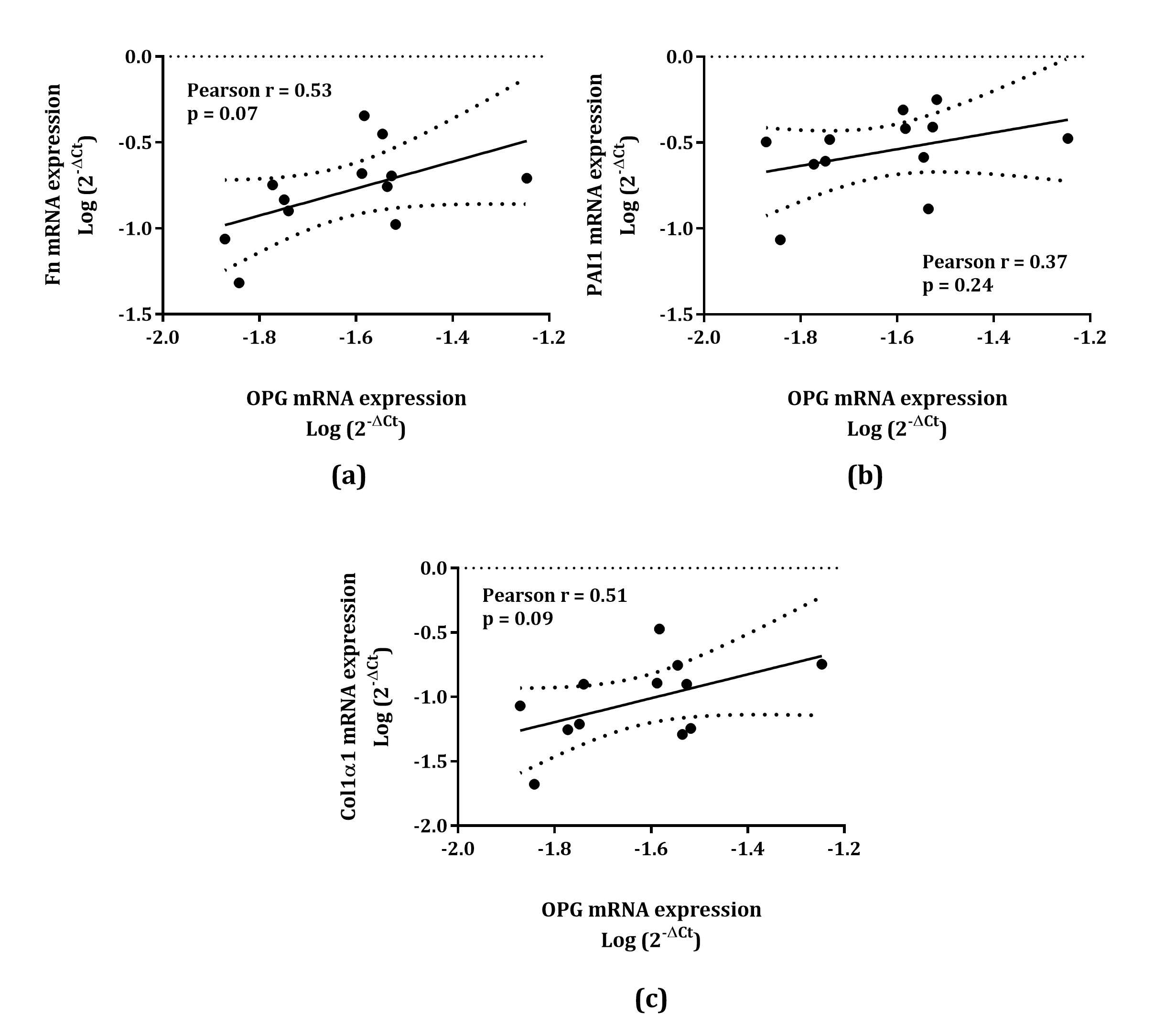

Supplement: Supplementary file 4 — Supplementary file4 (TIF 482 KB) [file 408_2024_691_MOESM4_ESM.tif]
